# Functional Role of AKNA: A Scoping Review

**DOI:** 10.3390/biom11111709

**Published:** 2021-11-17

**Authors:** Abrahán Ramírez-González, Joaquín Manzo-Merino, Carla Olbia Contreras-Ochoa, Margarita Bahena-Román, José Manasés Aguilar-Villaseñor, Alfredo Lagunas-Martínez, Yvonne Rosenstein, Vicente Madrid Marina, Kirvis Torres-Poveda

**Affiliations:** 1 Center for Research on Infectious Diseases, Instituto Nacional de Salud Pública, Cuernavaca 62100, Mexico; abraham-896@hotmail.com (A.R.-G.); ccontreras@insp.mx (C.O.C.-O.); mbahena@insp.mx (M.B.-R.); alagunas@insp.mx (A.L.-M.); vmarina@insp.mx (V.M.M.); 2 Department of Basic Research, Instituto Nacional de Cancerología, Mexico City 14080, Mexico; jmanzomerino@gmail.com; 3 Consejo Nacional de Ciencia y Tecnología (CONACyT)-Instituto Nacional de Cancerología, Mexico City 03940, Mexico; 4 Centro Nacional para la Salud de la Infancia y la Adolescencia (CeNSIA)-Secretaría de Salud Federal, Mexico City 01480, Mexico; manases.qfb@gmail.com; 5 Departamento de Medicina Molecular y Bioprocesos, Instituto de Biotecnología, Universidad Nacional Autónoma de México, Mexico City 62210, Mexico; yvonne.rosenstein@ibt.unam.mx; 6 CONACyT-Instituto Nacional de Salud Pública, Cuernavaca 03940, Mexico

**Keywords:** AKNA, function, immune response, inflammation, autoimmunity, cancer

## Abstract

Human *akna* encodes an AT-hook transcription factor whose expression participates in various cellular processes. We conducted a scoping review on the literature regarding the functional role of AKNA according to the evidence found in human and in vivo and in vitro models, stringently following the “PRISMA-ScR” statement recommendations. Methods: We undertook an independent PubMed literature search using the following search terms, AKNA OR AKNA ADJ gene OR AKNA protein, human OR AKNA ADJ functions. Observational and experimental articles were considered. The selected studies were categorized using a pre-determined data extraction form. A narrative summary of the evidence was produced. Results: AKNA modulates the expression of CD40 and CD40L genes in immune system cells. It is a negative regulator of inflammatory processes as evidenced by knockout mouse models and observational studies for several autoimmune and inflammatory diseases. Furthermore, AKNA contributes to the de-regulation of the immune system in cancer, and it has been proposed as a susceptibility genetic factor and biomarker in CC, GC, and HNSCC. Finally, AKNA regulates neurogenesis by destabilizing the microtubules dynamics. Conclusion: Our results provide evidence for the role of AKNA in various cellular processes, including immune response, inflammation, development, cancer, autoimmunity, and neurogenesis.

## 1. Introduction

Human *akna* is located on chromosome 9 (HGNC:24108), in band 32(9q32), within the FRA9E region of chromosome 9q32 [1], a common fragile site (CFS) linked to loss-of-function mutations that often lead to inflammatory and neoplastic diseases [2,3,4]. The 61-kb long *akna* gene contains 24 exons that encode nine different transcripts as the result of alternative promoter usage, splicing, and two polyadenylation sites [1,5]. AKNA contains a 9-amino-acid sequence (RTRGRPADS) that satisfies the consensus requirements of an AT-hook DNA-binding motif. AKNA also possesses three PEST regions associated with proteolysis [5]. The 69-kDa F1 isoform was the first to be described, and it is one of the few functionally tested [1,6]. It contains an N- and a C-terminus AT-hook domain, which enable the protein to bind to AT-rich DNA regions.

The human *akna* gene encodes a transcription factor present in the germinal center of secondary lymphoid organs and immune system cells (such as B and T cells), natural killer, and dendritic cells [5]. It has been demonstrated that some transcripts of AKNA are expressed in a tissue-specific manner, reflecting its functional diversity. AKNA has been reported to regulate multiple processes, but its functional role is not entirely clear. We performed a scoping review whose purpose was generally “to map the body of literature on a topic area” [7], in our case, the analysis of the functional role of AKNA.

## 2. Materials and Methods

### 2.1. Search Strategy

The review’s PICO (participants, intervention, comparison, outcome) question was: “What is the functional role of AKNA according to the evidence found in human subjects, as well as in vivo and in vitro models?”, in which P = AKNA protein, I = human, C = in vivo and in vitro models, and O = functional role. To address this question, we conducted a scoping review following the “PRISMA-ScR” statement recommendations (Table S1) [8].

A search in the MEDLINE database was performed through the PubMed database browser with a text formed by combining the following terms, “AKNA”, “AKNA gene”, “AKNA protein, human”, and “AKNA functions”. A Boolean operator (“OR”) was applied to link search terms to the research question. The search strategy used to locate studies in the primary electronic database was AKNA OR AKNA ADJ gene OR AKNA protein, human [MeSH terms] OR AKNA ADJ functions. The scope of the computerized literature search was expanded according to the reference lists of retrieved articles.

### 2.2. Study Selection

All searched articles were evaluated for their eligibility to be included in the review. First, the studies were evaluated by reading their titles/abstracts. Papers considered as relevant after reading their titles and abstracts were selected for further evaluation by reading their full text. Nine authors screened the original articles manually, and two of them were content reviewers, as specialists in the area (VMM and KTP). After the full-text evaluation, 21 papers were considered as eligible and were included in this scoping review.

### 2.3. Eligibility Criteria

All retrieved primary studies were further reviewed and checked for their eligibility to be included in the scoping review based on the criteria listed below.

### 2.4. Inclusion Criteria

#### 2.4.1. Study Design

Experimental studies (in vivo and in vitro) as well as observational studies (cross-sectional and case-control studies) with original data reporting the functional role of AKNA were considered.

#### 2.4.2. Language

The search was performed with a restriction of language of the full text to English.

#### 2.4.3. Publication Issue

Publications available online from 2000 (first report referring AKNA) to December 2020 were considered. The evidence included in this scoping review was mainly generated in the last five years, a period in which its functional role has been studied mostly.

### 2.5. Exclusion Criteria

During the full-text evaluation of the articles, we considered the study model, study design, study settings, paper quality, and outcomes of interest. Papers with a low-quality score and studies not reporting about the functional role of AKNA were excluded.

### 2.6. Data Extraction

Two authors (ARG, KTP) independently extracted all relevant data using a data extraction form built with Microsoft Excel (Microsoft Co., Redmond, WA, USA). The data extraction form included the name of the first author for each study, publication year, study model, study design, purpose of the study, sample size, methodology, relevant results, and proposed functional role for AKNA. Each section was populated in a single column, while rows were filled with data from each primary study. Any disagreements during data extraction were identified and resolved through discussion.

### 2.7. Outcome Measurement

Any functional role proposed for AKNA was explored and considered as an outcome.

### 2.8. Quality Assessment

The quality of the primary studies included in this scoping review was assessed using the STROBE scale for observational studies quality assessment [9]. To assess the quality of a study, an 11-item specific scale, based on the Strengthening the Reporting of Observational Studies in Epidemiology (STROBE) principles, was developed by consensus among the nine authors. Each item was categorized considering the introduction, methods, results, and discussion. The items covered study design, recruitment, and description of participants, as well as global quality (Table 1). The score was expressed in arbitrary units (a.u.) ranging from 0 to 22 (lower to higher quality). ARG and KTP independently evaluated the quality, and any disagreement was resolved after discussion.

The quality of in vivo primary studies that were included in this scoping review was assessed using the Animal Research: Reporting in vivo Experiments (ARRIVE) guidelines, a checklist of 20 items describing the minimum information that scientific publications reporting research using animals should include. This includes the number and specific characteristics of animals used (including species, strain, sex, and genetic background); details of housing and husbandry; the experimental, statistical, and analytical methods (including details of methods used to reduce bias such as randomization and blinding) (Table 2) [10].

The assessment of in vitro studies included in this scoping review considered their quality as evaluated by the Consolidated Standards of Reporting Trials (CONSORT) scale, modified to assess the quality of in vitro trials [19]. This modified CONSORT scale consists of 14 items that assess the quality of studies in terms of the abstract, introduction, methods, results, and discussion sections, along with other information of interest such as financial support received or access to a description of the test protocol employed. The items included in an article were marked with an asterisk; a blank box indicates that the item was not mentioned in the text (Table 3).

### 2.9. Synthesis of Results

The functional roles of AKNA emerging from the included data were analyzed and discussed within the research team. Descriptive functional role analyses are presented as narrative summaries, given the heterogeneity of the literature. Narrative summary is a methodology that may involve a simple recounting and description of findings to produce evidence [21].

### 2.10. Statistical Analysis

The data were compiled in a single spreadsheet and imported into Microsoft Excel 2019 (Microsoft Co., Redmond, WA, USA) for validation and coding. Fields allowing string values were examined for implausible values. The data were then exported into STATA version 14 (StataCorp, College Station, TX, USA) for analysis. Descriptive statistics were calculated to summarize the data. Frequencies and percentages were utilized to describe nominal data.

## 3. Results

### 3.1. Included Studies

In the first stage of our literature search, 38 articles were retrieved and considered as eligible after evaluating their title and abstract. According to the inclusion criteria (study model, study design, study settings, paper quality, outcomes of interest), 14 were excluded, and the remaining 24 articles were considered for a further full-text evaluation. After a full-text reading, three articles were excluded as they were literature reviews.

**Figure 1:**
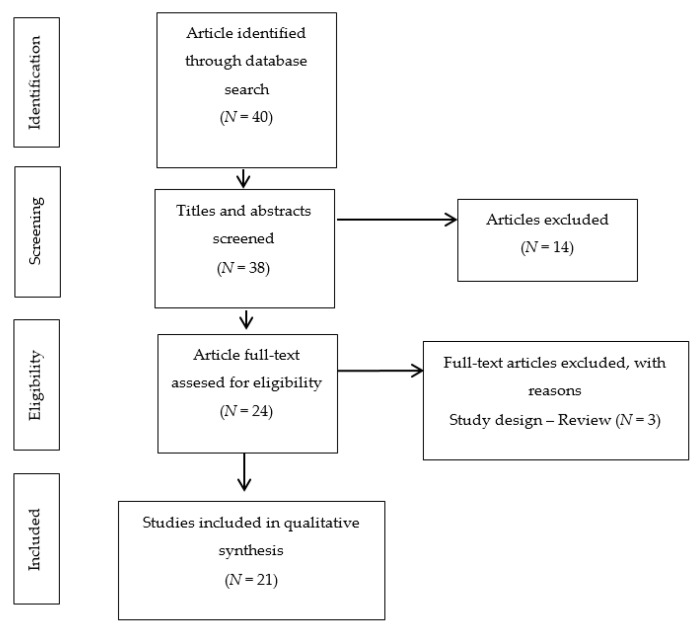
Flow diagram of the search process according to PRISMA-ScR. PRISMA Extension for Scoping Reviews.

In total, 21 papers were found as eligible to be included in this scoping review Figure 1 shows 10 observational studies (five cross-sectional studies [6,22,23,24,25] and five cases and controls studies [26,27,28,29,30]) and 11 experimental studies (five in vivo [11,12,13,14,15], three in vitro [1,5,20], and three studies featuring both in vitro and in vivo assays [16,17,18]).

#### 3.1.1. Quality Assessment of Included Studies

The mean (±SD) quality of the 10 selected observational studies was 20 (±1.8) a.u. (range 17 to 22), indicating a satisfactory level (Table 4).

Table 2 summarizes the quality assessment using the Animal Research: Reporting in vivo Experiments (ARRIVE) guidelines for the five in vivo studies and the three experimental studies featuring both in vitro and in vivo assays. A score of 0 indicates that the criterion is not reported or performed, whereas 1 indicates that the criterion is reported or performed. The mean quality assessment score of the included studies was 15 out of 20 points. All studies included an abstract, objectives, and background explaining the experimental approach, the study design for each experiment, the experimental procedures, details of the animals used and their source, experimental outcomes and estimation, scientific implications of the results, and their relevance to human biology. Most studies reported ethical approval statements (75), details of the statistical methods (62%), details of animal housing and husbandry conditions, randomizations or matching of experimental groups, and the numbers of animals (37%). Only 12% of the studies reported the details of sample size calculation, adverse events, and funding.

The quality of the in vitro experimental studies selected was evaluated by the CONSORT scale. Most of them (83%) were considered of moderate quality, as all of them met about 7 items out of 14. Table 3 shows the results of the CONSORT scale. All works included a complete abstract, an introduction describing the study’s scientific background and purpose, objectives and/or hypothesis, an accurate description of the results obtained, and sources of funding (items 1, 2a, 2b, 4, 11, and 13). The statistical methods used were described in five works (item 10). Only one article considered the limitations of the trial (item 12). None of the studies assessed met any of the other criteria that should be included in in vitro experimental trial studies (items 3, 5, 6, 7, 8, 9, and 14).

#### 3.1.2. General Characteristics of Included Studies

The 10 observational studies selected included a total of 4354 participants, with a relatively large sample size (>100) in two studies (Table 5). In four of the five studies related to infection-associated cancer, cancer was the only disease investigated: cervical cancer (CC) [6,27]; gastric cancer (GC) [25], and head and neck squamous cell carcinoma (HNSCC) [22]. Considering the number of individuals included, the most widely studied disease related to AKNA was degenerative knee osteoarthritis (KOA) (57.4% of participants in two studies) [28,30]. Two studies investigated the participation of AKNA in other autoimmune diseases, pSS (progressive systemic sclerosis) [29], and VKH (Vogt–Koyanagi–Harada disease) [26], representing 6% of all participants, and one publication evaluated disorders associated to chronic respiratory infectious diseases (PCD, primary ciliary dyskinesia) [24].

Regarding the five in vivo studies (Table 5), two independent gene-targeting mouse models engineered to assess the in vivo physiological significance of the AKNA were evaluated, a) *akna* knockout (KO) mice with deletion of the putative C-terminus AT-hook-like motif sequence of AKNA, and b) AKNA-KO with disruption of AKNA’s exon 3 (AKNA KO2) [11]. In either scenario, the resulting mice were smaller and weaker than WT; most of them died by day 10 of neonatal age. Causes of death included acute inflammatory reactions and alveolar destruction. Acute inflammation appeared to be characterized by a systemic neutrophil mobilization, alveolar infiltration, concerted activation of neutrophil-specific chemokines and cytokines, and the presence of extracellular matrix-remodeling enzymes. This study provided experimental support to the hypothesis that AKNA participates in the mechanisms that regulate the magnitude of inflammatory responses to pathogens [11].

The study by Suram et al. screened for genes that participate in the Candida albicans-stimulated response of mouse resident peritoneal macrophages downstream of cytosolic PLA_2_α (cPLA_2_α), the activation of which results in the production of arachidonic acid production and the release of eicosanoids and other bioactive lipids that modulate inflammation. When comparing the transcriptome of cPLA_2_α^+/+^ and cPLA_2_α^−/−^ macrophages, *akna* was one of the pro-inflammatory genes expressed at lower levels in the cPLA_2_α)^+/+^ macrophages with respect to cPLA_2_α^−/−^ cells, along with TNFα, Cx3cl1, CD40, CCL5, Csf1, Edn1, CxCr7, Irf1, Irf4, IFNγ, and several IFNγ-inducible GTPases [12], suggesting a role for AKNA in the regulation of host defense response and inflammation-related genes.

The study by Piulats et al. in nude mice was designed to evaluate chromosomal and genetic alterations in cisplatin-sensitive and cisplatin-resistant human testicular germ cell tumors orthoxenografts. The study revealed a clinical correlation between chromosomal rearrangements reflecting gains in the 9q32–q33.1 region in the cisplatin-refractory tumors and a poorer overall survival. The expression profile of the sixty genes located at that genomic region showed that POLE3 and AKNA were the only two deregulated genes in the cisplatin-resistant tumors harboring the 9q32–q33.1 gain, with POLE3 being upregulated and AKNA downregulated [13], underscoring a role for AKNA and potentially other A-T hook DNA-binding proteins in the cisplatin-resistant phenotype.

The work published by Hug et al. identified AKNA as the underlying causative genetic defect of recurrent pulmonary disease in rough collie dogs. The genomic sequence in one affected dog revealed a 4-bp deletion, c.2717_2720delACAG in the *akna* gene, resulting in the introduction of a premature stop codon, thus identifying AKNA as the causative variant for this inherited, autosomal recessive disease [14]. Although no germline coding variants of AKNA have been described to date in humans, and no individuals carrying loss-of-functions variants have been reported, this result and the report on the AKNA-KO mice by Ma et al. [11] points at *akna* as a candidate gene to explore for human patients with unexplained recurrent inflammatory pulmonary disease, not to mention viral-induced pulmonary inflammation, such as that resulting of SARS-CoV-2 infection.

In the last in vivo study included herein, while searching for causal gene polymorphisms related to or in strong linkage disequilibrium with racing performance in quarter horses, Pereira et al. analyzed exomes and UTR sequences in regions associated to a higher racing performance previously identified by a GWAS study. The *akna* gene was one of the nine new candidate genes identified in one of the three regions of interest for racing performance, with a deletion provoking a shift of the reading frame [15].

Two of the three in vitro experimental studies that were selected (Table 6) are pioneering works related to the discovery of the *akna* gene and its cellular functions [1,5]. Human AKNA has been characterized as an AT-hook nuclear protein that directly binds the AT-rich regulatory elements of the CD40 and CD40 ligand (CD40L-CD154) promoters, modulating their expression, and hence, B-cell differentiation, establishing the role of AKNA as a key transcription factor in immune regulation [5]. That same publication showed that AKNA mRNA was predominantly expressed in lymphoid organs, including the spleen and lymph nodes as well as peripheral blood leukocytes, natural killer cells, and dendritic cells [5]. At least nine distinct transcripts of AKNA have been identified, some of which are expressed in a tissue-specific manner (thymus and/or tonsils) and can be post-translationally modified [1], raising the possibility that different cells may preferentially express one isoform and/or that different circumstances may dictate the preference for one isoform. Altogether, these data suggest an important role for AKNA in the regulation of immune function, particularly through signaling resulting of the CD40-CD40L interaction.

When investigating the mechanism by which T cells from donors treated with the granulocyte colony-stimulating factor (G-CSF) show a reduced capacity to induce GVHD, a study featuring both in vitro and in vivo assays [16] (Table 6) reported that the expression of AKNA mRNA was significantly enhanced in CD4+CD25+ Treg cells. This study revealed that, by regulating CD40L and CD40 expression (and thus CD40-CD40L interactions), AKNA participates in a previously unrecognized role of the CD40-CD40L pair, the generation and maintenance of nTreg cells.

The study by Liu et al. evaluated the role of AKNA in the inflammatory response and growth hormone (GH) deficiency resulting from the exposure to T-2 toxin, a trichothecene produced by several species of the *Fusarium* mold genus that contaminate livestock and poultry food, and poorly stored cereals in general [17]. After a previous study had revealed that the T-2 toxin induced the expression of inflammatory cytokines and inhibited AKNA mRNA expression, the work by Liu et al. investigated the T-2 toxin-induced signaling pathway that regulates AKNA mRNA expression and its effect on the secretion of inflammatory cytokines and the production of growth hormone in rats. The T-2 toxin induced pCREB and NF-κB/p-p65 direct binding to the AKNA promoter, inhibiting AKNA mRNA expression while relocating the existing AKNA molecules from the cytoplasm to the nucleus to regulate the expression of downstream genes that have an AT sequence, particularly pro-inflammatory cytokines [17]. This study provided new insights into the signaling pathway leading to AKNA expression, stressing the role of AKNA in the inflammatory response. In addition, this work raised the concern that chronic exposure to low doses of this mycotoxin may directly impact human health.

Another in vitro study brings evidence related to the possible role of AKNA in the development of cervical cancer [20]. The E6 oncoprotein from high-risk HPVs 16 and 18 was found to interact with and downregulate AKNA, as well as CD40, in a proteasome-dependent manner. Furthermore, p53 was shown to interact with AKNA, promoting the expression of AKNA, leading the authors to propose that by downregulating the expression of CD40, the E6/p53/AKNA axis de-regulates the immune system, favoring tumor growth [20]. Whether AKNA acts as a tumor suppressor in the cervix epithelial cells, potentially contributing to epithelial-mesenchymal transition and cell transformation as it was recently documented for gastric cancer [25] and suggested by the Camargo et al. study, remains to be investigated [18].

While searching the developing mouse cerebral cortex for candidate regulators of the generation and expansion of the subventricular zone (SVZ), a recent study found that AKNA is necessary and sufficient to organize centrosomal microtubules, promoting their nucleation and growth to mediate the formation of the subventricular zone. Moreover, high AKNA levels regulate the exit of neural cells from the subventricular zone, revealing an unsuspected pivotal role of AKNA in the centrosomal microtubule organization and the formation of the subventricular zone, a zone in the forebrain particularly important during intrauterine development and where the majority of neural cells (neurons and microglia) originate [18]. Furthermore, data presented in that study suggest that by regulating the centrosomal microtubule organization, AKNA may participate in the epithelial-to-mesenchymal (EMT) transition in tumor epithelial cells, a possibility also raised by Wang et al. [25].

#### 3.1.3. Main Findings

Table 7 shows the current status of the main findings reported so far in the scientific literature regarding the functional role of AKNA, presented in chronological order of publication.

## 4. Discussion

This scoping review provides relevant considerations on the role of AKNA in different cellular processes. Despite having conducted a comprehensive search in available databases, only a small number of relevant articles meeting the terms of eligibility were identified, mainly those associated to the regulation of the inflammatory response. Interestingly, the terms of the screening allowed us to retrieve several publications highlighting new roles for AKNA in organs or tissues other than the immune system, considerably widening the range of action of AKNA. Although AKNA was first discovered as a maturation molecule of B lymphocytes [5], several studies revealed new functions for this protein in the immune system but also in pulmonary diseases, cancer, and neurogenesis, consistent with the fact that the lifespan of AKNA-KO mice is only a few days [11]. Overall, the body of information we evaluated underscores the important role of AKNA in health and disease.

Three of the publications using animal models yielded strong experimental evidence about the relationship of AKNA with inflammatory disease, stressing the immune-related role of AKNA. The results presented by Ma et al. and Suram et al. highlight the role of AKNA in regulating the intensity of inflammatory responses to pathogens, since AKNA-KO mice presented an exacerbated inflammatory response leading to tissue destruction due to neutrophil infiltration and the expression of pro-inflammatory mediators, such as interleukin-1β and interferon-γ, particularly in the lung [11,12]. The deregulated inflammatory phenotype of AKNA-KO mice is consistent with the results presented by Hug et al., where AKNA is proposed to function as a negative regulator of the immune response in a hereditary recurrent pulmonary disease in rough collie dogs [14]. In addition, to contribute to our understanding of the role of AKNA, those animal models provide valuable platforms to continue exploring the functions of AKNA in the future.

The different in vitro approaches evaluated in this review provided substantial knowledge on *akna* regulation, its target genes, and the signaling pathways controlling the transcription of AKNA. The multiple AKNA transcripts identified in different immune cell types (mature tonsil lymphocytes or thymic lymphocytes) suggest a potential differential expression of AKNA with different targets and functions, depending on the cell type or isoform [1]. Particularly, AKNA binding to AT-rich regions on the CD40 and CD40L promoters has been shown to regulate the expression of these co-receptors, modulating several immune cells functions, including the generation and maintenance of nTreg cells, revealing new functions for CD40-CD40L and AKNA [5,16]. Regarding the regulation of AKNA expression, the findings presented by Liu et al., identified pCREB and p-p65/NF-kB as two transcription factors that inhibit AKNA expression [17]. As the expression of AKNA in different tissues and cells evidences a role for this protein beyond the immune system, understanding the molecular mechanisms that regulate AKNA expression in different tissues opens a field for future research. One avenue to investigate would be whether pCREB and p-p65 NF-kB are regulated by a signaling pathway downstream of the cPLA_2_α activity, as demonstrated by Suram et al. [12].

Most interestingly, several studies revealed multiple and new functions for AKNA. While searching for polymorphisms associated with racing performance in racing horses, a deletion causing a shift of the reading frame of *akna* was identified [15]. Although the authors did not further characterize whether this SNP results in a premature stop codon or a truncated protein, this finding raises questions about the precise function of AKNA in modulating the different systems (musculoskeletal, cardiovascular, metabolic, nervous) involved in racing performance.

In the search for factors that regulate the maintenance or differentiation of neural stem cells, the work by Camargo et al. demonstrated that AKNA is essential to organize the centrosomal microtubules in neural stem cells and regulate their stemness or differentiation capacity. Low levels of AKNA promote the stem cells to remain in the stem cell niche, whereas higher levels of expression favor their detachment and migration out of the stem cell niche to differentiate, a phenomenon known as delamination [18]. Delamination processes are primarily mediated by the effect of AKNA on microtubule dynamics by destabilizing apical microtubule-actin-AJ complexes, which promote the constriction of apical end feet [18,31]. Consistent with this new role of AKNA in neurogenesis favoring the delamination processes [31], a frameshift variant in exon 12 of *akna* was recently reported to co-segregate with microcephaly, a prenatal condition with varying degrees of intellectual disability. The frameshift was predicted to result in a truncated C-terminus [32], shown by Camargo et al. to be indispensable for the localization of AKNA in the centrosome and for normal brain development [18].

The role of AKNA in cancer is coming to light. A study aiming to evaluate chromosomal and genetic alterations in cisplatin-sensitive and cisplatin-resistant human testicular germ cell tumors identified a downregulation of AKNA in the cisplatin-resistant tumors with chromosomal rearrangements in the 9q32-q33.1 region [24]. This study suggested a role for this transcription factor in downregulating the expression of molecules associated with the cisplatin-sensitive phenotype. Similarly, single nucleotide polymorphisms (SNPs) in the AT-binding region of AKNA are associated with a higher risk to develop cervical cancer [6], suggesting that impairing the function of AKNA favors the acquisition of a transformed phenotype. Along the same line, the publications by Martinez-Nava et al. [27], Manzo-Merino et al. [20], Wang et al. [25], and recently Liao et al. [33] on bladder cancer indicate that AKNA participates and is somehow downregulated in different cancer types with an epithelial origin, revealing a protective role for AKNA in cell transformation. Interestingly, the study by Camargo et al. reported that AKNA mRNA levels were regulated by Sox4, a master regulator of epithelial-mesenchymal transition, tumor growth, and metastasis, consistent with all those previous publications describing a role for AKNA in cancer.

**Figure 2:**
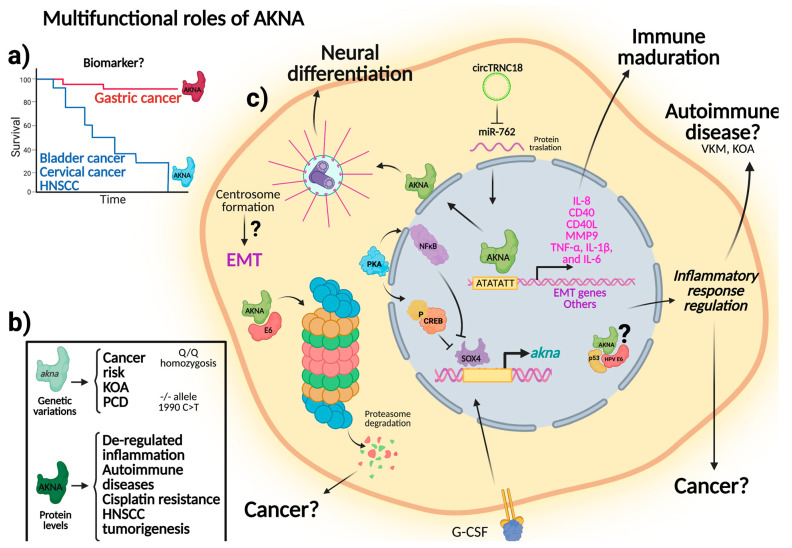
Multifunctional roles of AKNA. The impact of either protein levels or variations on the encoding AKNA gene have been studied in different models. A summary of the known activities of AKNA is depicted. (**a**) AKNA expression levels have been proposed as potential prognostic factors in different types of cancer; (**b**) variations in the AKNA sequence are associated to an increased risk for developing cancer, KOA, PCD. AKNA protein levels are involved in immune de-regulation, autoimmune diseases, cisplatin resistance, and HNSCC tumorigenesis; (**c**) molecular functions of AKNA are involved in the regulation of the inflammatory phenotype due to the ability of AKNA to promote cytokine gene expression, which in turn produces an aberrant immune regulation. AKNA has also been associated with cancer development, neural differentiation, and autoimmune disease, but the precise mechanisms underlying these processes remain unknown. The potential implications of AKNA in different cellular processes are indicated. HNSCC: head and neck squamous cell carcinoma; KOA: knee osteoarthritis; PCD: primary ciliary dyskinesia.

The research to evaluate the association between AKNA polymorphisms and the occurrence of KOA, PCD, pSS, and inflammatory pulmonary disease in dogs evidenced a probable causality link between AKNA and these diseases. Likewise, prediction models in cases of sepsis have proposed AKNA as a gene associated with immunosuppression and a high mortality rate [34]. Furthermore, from a broader perspective, these data offer insights into the multifunctional role of AKNA (Figure 2), an incentive to decipher the different actions of AKNA in various cellular scenarios.

One of the most important limitations that needs to be addressed in the present scoping review is the small number of relevant articles that were identified, which might be explained by the novelty of this molecule and the difficulties involved in setting up either in vitro or in vivo studies to further demonstrate a role for AKNA. Thus, there are limited data proving the functional role of AKNA, since only a few work groups have focused on depicting the implications of AKNA loss in development and inflammation. Additionally, when analyzing the quality of the studies, most of them were scored with moderate quality, since it is hard for this type of in vitro research to meet high-quality criteria.

## 5. Conclusions

This scoping review gathers relevant scientific evidence on the role of AKNA in various cellular processes, primarily associated to:

i. Inflammation: Expression of proinflammatory cytokines.
ii. Immune response: CD40 and CD40L regulation and B-cell maturation.
iii. Development: Centrosomal microtubule reorganization and delamination of certain types of neural stem cells by an EMT-like process.
iv. Molecular mechanisms associated to pathologic processes (gastric and cervical cancer, Vogt-Koyanagi-Harada syndrome, knee osteoarthritis, primary Sjögren’s syndrome, primary ciliary dyskinesia, head and neck squamous cell carcinoma, and acute lymphoblastic leukemia) being a key transcription factor that regulates genes involved in immunity, inflammation, and cancer.

Investigating the potential role of different AKNA transcripts and the signaling pathways that regulate its expression, as well as identifying its molecular partners in immune cells, neural stem cells, cancer cells, or other cell types, will open new avenues for research and allow us to better understand the role of AKNA in cell proliferation and differentiation, both in health and disease.

## Figures and Tables

**Table 1:** Quality analysis form used in the scoping review for observational studies.

Section	Question
Introduction	Q1 Is the scientific context clearly explained?
	Q2 Are the objectives clearly stated?
Methods	Q3 What is the design of the study? (1 cross-sectional; 2 case and control)
	Q4 Are inclusion criteria and the selection of participants clearly explained? Q5 Sample size (0 if <20, 1 if 20–100, 2 if >100) Q6 Is the method (validity) described? Q7 Are statistical analyses appropriate?
Results	Q8 Are subjects’ characteristics described?
	Q9 Are the results interpretable?
Discussion	Q10 Are the study findings discussed with related studies published in the literature?
	Q11 Are study limitations discussed?

For questions 1,2,4,6, 7,8, 9, 10, and 11 the scoring was as follows, 0, no description; 1, limited description; 2, good description.

**Table 2:** Quality of articles assessed with the ARRIVE guideline for in vivo studies.

Studies	1	2	3	4	5	6	7	8	9	10	11	12	13	14	15	16	17	18	19	20	Score
Ma et al. (2011) [11]	*	*	*	*		*	*	*	*		*	*	*	*	*	*	*	*	*		17
Suram et al. (2013) [12]	*	*	*	*	*	*	*	*				*		*		*		*	*		13
Piulats et al. (2018) [13]	*	*	*	*	*	*	*	*				*	*	*		*		*	*		14
Hug et al. (2019) [14]	*	*	*	*	*	*	*	*				*		*		*		*	*	*	14
Pereira et al. (2019) [15]	*	*	*	*	*	*	*	*				*		*	*	*		*	*		14
MacDonald et al. (2014) [16]	*	*	*	*		*	*	*				*	*	*		*		*	*		13
Liu et al. (2017) [17]	*	*	*	*	*	*	*	*	*		*	*	*	*		*		*	*		17
Camargo et al. (2019) [18]	*	*	*	*	*	*	*	*	*	*	*	*	*	*	*	*		*	*		18

The first 5 studies performed in vivo assays only; the 3 remaining studies featured both in vitro and in vivo assays. Criteria: (1) A title that provides a concise description of the content of the article; (2) A structured abstract with details of the animal species or strain of animal used; (3) A background that explains the experimental approach; (4) Objectives that describe the specific hypotheses being tested; (5) A methods section with an ethical statement for the care and use of ani-mals that cover the research; (6) A study design described for each experiment (number of experimental and control groups, steps taken to minimize bias, experimental units); (7) Experimental procedures described (how, when, where, and why for each experiment and each experimental group, including controls); (8) Experimental animals (details of the ani-mals used and their source); (9) Housing and husbandry (details and welfare-related assessment and interventions); (10) Sample size (number of animals in each experiment and each experimental group, the calculation method used and num-ber of replicates); (11) Allocating animals to experimental groups (randomizations or matching, order of treatment and assessment); (12) Experimental outcomes (primary and secondary outcomes assessed); (13) Statistical methods (unit of anal-ysis and details of the methods used); (14) In the results section, baseline data for each experimental unit before treatment); (15) Numbers analyzed (number of animals in each group included in each analysis); (16) Outcomes and estimation (results for each analysis with a measure of precision); (17) Adverse events (details in each experimental group); (18) In the discus-sion section, scientific implications (interpretation of the results, limitations of the study); (19) Generalizability (translation of the findings and relevance to human biology); (20) Funding (sources and role of the funder). * Indicates the fulfillment of the criterion.

**Table 3:** Quality of articles assessed with the modified CONSORT scale for in vitro studies.

Studies	1	2a	2b	3	4	5	6	7	8	9	10	11	12	13	14	Score
Siddiqa et al. (2001) [5]	*	*	*		*							*		*		6
Sims et al. 2005 [1]	*	*	*		*						*	*		*		6
Manzo-Merino et al. (2018) [20]	*	*	*		*						*	*		*		7
MacDonald et al. (2014) [16]	*	*	*		*						*	*	*	*		8
Liu et al. (2017) [17]	*	*	*		*						*	*		*		7
Camargo et al. (2019) [18]	*	*	*		*						*	*		*		7

The first 3 studies performed in vitro assays only; the 3 remaining studies featured both in vitro and in vivo assays. Criteria: (1) Structured abstract; (2a) scientific background; (2b) objectives and/or hypothesis; (3) intervention; (4) the way in which and the moment when outcomes were evaluated; (5) sample size determination; (6) method used to generate a random allocation sequence; (7) mechanism used to generate a random allocation sequence; (8) who generated the random allocation sequence; (9) who was blinded for random allocation and how; (10) statistical methods for comparing outcomes; (11) precision of the results obtained; (12) study limitations; (13) sources of funding; (14) access provided to study the protocol. * Indicates the fulfillment of the criterion.

**Table 4:** Quality assessment analysis using STROBE scale for observational studies.

Studies	Q1	Q2	Q3	Q4	Q5	Q6	Q7	Q8	Q9	Q10	Q11	Total
Perales et al. (2010) [6]	2	2	1	2	2	2	2	2	2	2	1	20
Mao et al. (2011) [26]	2	2	2	2	1	2	2	2	2	2	1	20
Martínez et al. (2015) [27]	2	2	2	2	2	2	2	2	2	2	2	22
Chen et al. (2015) [22]	2	2	1	1	1	2	2	2	2	2	0	17
Hernández et al. (2017) [29]	2	2	2	2	2	2	2	2	2	2	2	22
Martínez et al. (2018) [28]	2	2	2	2	2	2	2	2	2	2	2	22
Song et al. (2019) [23]	2	2	1	2	2	2	2	2	2	2	2	21
Shamseldin et al. (2020) [24]	2	2	1	1	1	2	2	2	2	2	2	19
Wang et al. (2020) [25]	2	2	1	1	1	2	2	2	2	2	1	18
Zhao et al. (2020) [30]	2	2	2	2	2	2	2	2	2	2	2	22

**Table 5:** Characteristics of the selected observational studies selected for the scoping review.

Author, Year	Study Design	No. of Participants	Principal Study Criteria	Main Finding
Perales et al. (2010) [6]	Cross-sectional	47 human papillomavirus (HPV)-positive biopsies from Mexican women diagnosed with squamous intraepithelial lesion (SIL) (N = 21) or CC (cervical cancer) (N = 26) and samples of apparently healthy women (N = 50) non cervical lesion (NCL) and with HPV-negative status	To investigate the allelic frequency of arginine (R)-glutamine (Q) as potential risk factors for HPV-associated CC	AKNA Q/Q homozygosis is a risk factor for CC associated with HPV infection
Mao et al. (2011) [26]	Cases and controls	N = 21, 10 cases and 11 controls	To identify differentially expressed membrane proteins in patients with active Vogt-Koyanagi-Harada syndrome (VKH) and controls	A possible regulation of the CD18 molecule (integrin B2, which participates in cell adhesion, neutrophil chemotaxis, and cell signaling) was found, and a role in apoptosis of CD4 + T cells and a decreased level of AKNA expression could be observed, along with a possible down-regulation in the expression of CD40L in T cells
Martínez et al. (2015) [27]	Cross-sectional	420 HPV^+^ women, 109 NCL, 149 SIL, and 162 CC	To assess the association of single nucleotide polymorphisms (SNPs) of AKNA F1 isoform promoter region with SIL and CC, as well as their effect on *akna* mRNA expression levels in peripheral blood mononuclear cell (PBMCs) in both stages of the disease, by simultaneously measuring the number of transcripts from each allele and the feature known as allelic expression imbalance (AEI)	Two polymorphisms were associated with SIL and CC, and an association between high *akna* expression levels and CC and SIL was identified, although its effects differed in each disease stage. To show the potential existence of a cis-acting polymorphism, the *akna* allelic expression imbalance was evaluated for the alleles of the−1372C4A polymorphism, and the number of transcripts derived from the A allele was found to be significantly higher than those from the C allele
Chen et al. (2015) [22]	Cross-sectional	Five pediatric acute lymphoblastic leukemia (ALL) patients and validation cohorts with non-recurrent high hyperdiploid ALL (N = 6), recurrent ETV6-RUNX1-positive ALL (N = 7), non-recurrent Down-syndrome associated (N = 6), and TCF3-PBX1-rearranged (N = 5) ALL	To identify relapse-associated mutations in patients with hyperdiploid acute lymphoblastic leukemia by sequencing	Somatic mutations were also detected in signaling molecules (AKNA, PPP1R3C, NLRP4, GLIS1, BAX) involved in B cell the differentiation, proliferation and in the relapse samples of recurrent hyperdiploid ALL
Hernández et al. (2017) [29]	Cases and controls	110 patients with primary Sjögren’s syndrome (pSS) and 141 ethnically matched healthy controls	To evaluate the allele and genotype frequencies of polymorphic sites of the HIF1A and AKNA genes in pSS	The HIF1A Pro582Ser (rs11549465) T allele and C/T genotype, as well as the AKNA−1372C>A (rs10817595) A/A genotype were identified as susceptibility genetic factors for pSS, conferring the former a decreased and the latter an increased risk of pSS in a Mexican mestizo population
Martínez et al. (2018) [28]	Cases and controls	81 knee osteoarthritis (KOA) patients and 140 healthy controls	To assess the potential association of AKNA polymorphisms with KOA susceptibility in a Mexican population	Regulatory and coding polymorphisms of *akna* can influence the development of KOA
Song et al. (2019) [23]	Cross-sectional	250 samples from head and neck squamous cell carcinoma (HNSCC) patients	To predict an intrinsic relationship or correlation between the *akna* expressions and HNSCC; to validate these hub genes (genes with a high correlation in candidate modules) in the Cancer Genome Atlas (TCGA) dataset to confirm that they have a biological significance in the tumorigenesis of HNSCC	*akna* is one of the 16 hub genes that play an important role in HNSCC tumorigenesis, and could be used as a biomarker in the future
Shamseldin et al. (2020) [24]	Cross-sectional	81 patients in 56 families	To identify pathogenic gene variants associated with primary ciliary dyskinesia (PCD)	Homozygous nonsense variation in *akna* 1990C>T:p.(Glna664*) was associated to PCD and AKNA is proposed as a novel candidate in a lung phenotype that overlaps clinically with PCD and a potentially multiciliated cell-specific role
Wang et al. (2020) [25]	Cross-sectional	32 fresh primary (gastric cancer) GC and 32 matched normal gastric epithelial tissues from patients with GC undergoing resection in the First Affiliated Hospital of China Medical University	To investigate the role of AKNA in GC	AKNA could act as a tumor suppressor by modulating epithelial-mesenchymal transition (EMT)-related pathways in GC. AKNA could serve as a potential biomarker and an effective target for GC diagnosis and therapy
Zhao et al. (2020) [30]	Cases and controls	2500 Han Chinese subjects comprising 824 KOA patients and 1676 controls	To investigate the association between the AKNA gene and susceptibility to KOA in a Han Chinese population	This study is the first to provide evidence of a potential link between the risk of KOA and *akna* gene polymorphism among subjects with Han Chinese ancestry

**Table 6:** Characteristics of the in vivo and in vitro experimental studies selected for the scoping review.

Author, Year	Study Model	Experimental Units	Principal Study Criteria	Main Finding
Ma et al. (2011) [11]	Wild type and AKNA-KO mice	AKNA-KO and AKNA-KO2 mice compared to wild-type mice	To provide experimental support to the hypothesis that AKNA expression plays an important role in the mechanisms that regulate the magnitude of inflammatory responses to pathogens	AKNA plays a role in mechanisms that regulate the magnitude of acute inflammatory responses by coordinately repressing genes involved in neutrophil activation, mobilization, and function. The authors hypothesize that the increased expression of cytokines, chemotactic factors, and MMP9 resulting from AKNA deficiency may indeed reflect a loss of the repressive function of the gene
Suram et al. (2013) [12]	Pathogen-free Balb/c mice	Cytosolic phospholipase A2 (cPLA2)α^−^^/^^−^ mice, TLR4 mutant mouse strain C3H/HeJ, control strain C3H/HeOuJ, TLR2^−^^/^^−^ (C57BL/6), MyD88^−^^/^^−^ mice (C57BL/6/129), MyD88^+/^^−^ C57BL/6/129 mice were crossed to produce MyD88^−^^/^^−^ mice and MyD88^+/+^ littermate controls, C57BL/6 control mice and Dectin-1^−^^/^^−^ mice (129sv/ev)	To investigate the functional consequences of cPLA2α activation and the effect of endogenously produced eicosanoids on gene expression in response to *C. albicans*	Genes for interferon regulatory factor (Irf)1 and Irf4 and AKNA were expressed at lower levels in A2α cPLA2α^+/+^ mouse peritoneal macrophages stimulated with *C. albicans* than in cPLA2α^−^^/^^−^ macrophages, suggesting a correlation between a low AKNA expression and CD40 expression level
Piulats et al. (2018) [13]	Murine model (nude mice)	One nude mouse strain	To investigate the genetic basis of cisplatin resistance, as the efficacy of 82 cisplatin-based chemotherapy in the treatment of distinct malignancies is often hampered by intrinsic or acquired drug resistance of tumor cells	*akna* was negatively regulated in cisplatin resistant tumors with gains of 9q32-q33.1
Hug et al. (2019) [14]	Rough collie dogs and dogs of various other breeds	88 rough collie dogs, 539 dogs of various other breeds as controls	To identify the genetic origin of recurrent pulmonary disease in rough collie dogs	A variant with a 4-bp deletion (c.2717_2720delACAG) in the *akna* gene was identified as a causative variant candidate for an inherited, autosomal recessive, recurrent inflammatory pulmonary disease in dogs. Genetic analysis showed a genotype-phenotype correlation of the AKNA variant with inflammatory pulmonary disease
Pereira et al. (2019) [15]	Horses	Two groups, high- vs. low-performance in racing; 360 specimens of racing quarter horses, 78 males and 282 females	To analyze exomes and UTRs in regions previously associated to racing performance by GWAS in quarter horse racehorses	AKNA was related to racing performance in the quarter horse breed, with a positive regulation of transcription from polymerase II RNA promoter
Siddiqa et al. (2001) [5]	Cells of the immune system, lymphoid and non-lymphoid tissues	Human B lymphocytes, lymphoid tissues, and other kinds of non-lymphoid tissues. The article does not indicate the number of experiments performed	To evaluate the role of the AT-hook transcription factor as a regulator of CD40 and CD40L gene expression	AKNA is a human nuclear protein that contains multiple PEST protein-cleavage motifs, it is mainly expressed by B and T lymphocytes, NK, and dendritic cells. During B-lymphocyte differentiation, it is expressed by germinal center B lymphocytes. AKNA acts as an AT-hook protein that binds the A/T-rich regulatory elements of the promoters of CD40 and CD40L and coordinately regulates their expression
Sims et al. 2005 [1]	Human cell lines and tissues	Normal human mononuclear cells. Various cell lines including the PreB acute lymphocyte leukemia (PreB ALL) cell line Blin-1, Nalm 16, the Burkitt lymphoma cell line Raji, the T-cell line Jurkat, the erythroleukemia cell line K562, and the lymphoblastoid cell line JY. Primary B-lymphocytes purified from fresh tonsils. Tonsils or human thymus. The article does not indicate the number of experiments performed	To analyze the expression of multiple AKNA transcripts	Many of the AKNA transcripts originate from alternative splicing; others derive from differential polyadenylation and promoter usage. The alternative AKNA transcripts encode overlapping protein isoforms (p70 and p100), which can have a common function The AKNA PEST-dependent cleavage occurs in mature B cells and is required for CD40 upregulation. Multiple isoforms possibly expressed in a tissue-specific manner. The AKNA F1 and F2 isoform bind to the promoter of costimulatory molecules CD40 and CD40L (CD154)
Manzo-Merino et al. (2018) [20]	Human cell lines and tissues	HPV-positive cell lines, including SiHa (positive for HPV16) and HeLa (positive for HPV18), as well as the HPV-negative cell lines HaCaT and HEK293T. Primary human keratinocytes. Cervical tissue from 10 cases of hysterectomy for squamous cell carcinoma and four samples of normal cervical epithelium	To identify the effect of the HPV E6 oncoprotein on AKNA	AKNA promotes CD40 and IL-8 expression in keratinocytes
MacDonald et al. (2014) [16]	Murine model and cells	8–12 weeks-old mice. T cells purified of non-T cell splenocytes. Splenic CD3+CD4+GFP+ Tregs. Data pooled from two- and three-replicate experiments	To evaluate the effect of human granulocyte colony-stimulating factor (G-CSF) on the modification of T cell responses	AKNA is upregulated in Treg cells upon stimulation with G-CSF
Liu et al. (2017) [17]	Murine model and GH3 cell lines	16 specific pathogen-free female Wistar rats (*Rattus norvegicus*, 5–6-weeks-old). GH3 cells from the Cell Bank of the Academy of Sciences (Beijing, China). All experiments were performed at least in triplicate on three separate occasions	To evaluate the role of AKNA in the inflammatory response mediated by the T-2 toxin-induced and growth hormone deficiency	AKNA was found to be a key regulator of T-2 toxin-mediated growth hormone deficiency through a p-p65/p-CREB dependent mechanism
Camargo et al. (2019) [18]	Murine model and BAC-transgenic cell lines	Female and male mice at embryonic stages E9, E11, E13, E14, E15, and E18. 2–12 months-old C57BL/6J and RjHan:NMRI mice. Primary E14 cortical cells and Neuro2A, NMuMG, human iPS, A20, and Mpf cells. All experiments in this study, except proteomic analyses, were replicated multiple times with the same experimental protocol, followed by the same analysis	To search for candidate regulators of neural cell differentiation and the fate of cells	AKNA was found to be essential to organize centrosomal microtubules and promote their nucleation and growth, showing that, depending on the levels of AKNA, this protein controlled the delamination process in the formation of the subventricular zone, or the exit from the subventricular zone, revealing the critical role of AKNA in the organization of the centrosomal microtubules

The first five studies performed in vivo assays only; the following three studies performed in vitro assays only; the three remaining studies featured both in vitro and in vivo assays.

**Table 7:** Main findings on the functional role of the AKNA in the reported in literature.

Author, Year	Finding	Functional Interpretation
Siddiqa et al. (2001) [5]	Predominant expression of AKNA in secondary lymphoid organs	AKNA could be important in antigen-dependent immune responses
	AKNA is predominantly expressed by germinal center B lymphocytes	AKNA may be important in the physiology of germinal center reaction
	AKNA can bind CD40 regulatory elements and to AT-rich CD40L promoter elements and upregulates the expression of CD40L	AKNA is a key transcription factor that regulates the expression of the CD40-CD40L receptor/ligand pair highlighting the physiological significance of AKNA during immune responses
Sims et al. 2005 [1]	The AT-hook transcription factor AKNA is encoded by a single gene locus mapping to chromosome 9q32	The p70 and p100 isoforms of AKNA originating from distinct alternatively processed mRNA and translated from different translation-initiation sites exhibit similar functions, pointing at the role of AKNA in controlling the amplitude of the immune response
	AKNA expresses at least nine distinct transcripts, some of which are expressed in a tissue-specific manner	AKNA can be post-translationally processed to regulate the expression of CD40
Perales et al. (2010) [6]	AKNA Q/Q homozygosis is a risk factor for human papillomavirus (HPV)-associated cervical cancer (CC)	AKNA appears to be an important genetic factor associated with the risk of CC
Ma et al. (2011) [11]	*akna*^−/−^ mice die postnatally due to severe and destructive lung infections	AKNA expression plays an important role in the mechanisms that regulate the magnitude of inflammatory responses to pathogens
Mao et al. (2011) [26]	AKNA expression is diminished in CD4+ T cells from patients with the autoimmune disease Vogt-Koyanagi-Harada (VKH) syndrome	A role for AKNA in the pathogenesis of the autoimmune disease VKH syndrome is suggested
Suram et al. (2013) [12]	The AT-hook transcription factor AKNA is expressed at lower levels in cytosolic phospholipase A2α cytosolic phospholipase A2 (cPLA2)α^+/+^ mouse peritoneal macrophages stimulated with *C. albicans* than cPLA2α^−^^/^^−^ macrophages	AKNA came up as a molecule that dampens the inflammatory response downstream of the cPLA2α signaling elicited by *C. Albicans* in mouse peritoneal macrophages
MacDonald et al. (2014) [16]	Human granulocyte colony-stimulating factor (G-CSF) upregulated AKNA mRNA in nTreg cells	Although AKNA was reported to be expressed by CD4 T cells, no studies thus far have characterized the expression or function of AKNA in Tregs cells
	There is a significant impairment in Treg development and maintenance in CD40-deficient mice	A novel role for CD40-CD40L interactions in the generation and maintenance of Treg cells was demonstrated. Further investigations determine the mechanism by which CD40 signaling contributes to Treg cells biology are necessary
Martínez et al. (2015) [27]	(i) Significant negative association between squamous intraepithelial lesion (SIL) and CC with *akna* −392C4T and −1372C4A polymorphisms, (ii) significant association of *AKNA* expression levels at the cervix and in PBMC with CC diagnosis, and (iii) AKNA expression differs across SNP genotypes in CC patients	*akna* is a CC susceptibility genetic factor and the *akna* transcriptional regulation has a role in this disease
Chen et al. (2015) [22]	Somatic mutations in signaling molecules such as AKNA are involved in the differentiation, proliferation, and death of B cells in the relapse samples of recurrent hyperdiploid acute lymphoblastic leukemia (ALL)	Mutations found in *akna* may play key roles in the pathogenesis of recurrent disease in high hyperdiploid ALL
Hernández et al. (2017) [29]	The HIF1A Pro582Ser *T* allele and *C/T* genotype and *akna* −1372C > A polymorphism A/A genotype are proposed as genetic factors associated with primary Sjögren’s syndrome (pSS), in the Mexican mestizo population	AKNA function as a transcription regulator of CD40L and CD40 could be relevant in pSS pathology, in the Mexican population
Liu et al. (2017) [17]	AKNA is suggested to be a key regulator of T-2 toxin-mediated inflammation and growth hormone deficiency	AKNA has a significant role in T-2 toxin-induced inflammatory response and growth inhibition. The PKA/CREB and NF-κB pathway participate in the signaling regulating AKNA expression
Manzo-Merino et al. (2018) [20]	AKNA expression is lower in CC tissue than in normal tissues	Inherent AKNA deficiency in neoplastic epithelial cells, not found in immune cells that conform the tumoral inflammatory infiltrate
	The HPV E6 oncoprotein de-regulate AKNA and CD40 expression. This event involves the action of p53	The E6/p53/AKNA axis might play an important role in the de-regulation of the immune system during HR-HPV-induced carcinogenesis in CC promoting epithelial-mesenchymal transition (EMT)
	Restoration of AKNA levels in HeLa cells induces the expression of interleukin 8 (IL-8)	The inflammatory status promoted by HPV via p53/AKNA dampens immune response favoring tumor growth
Martínez et al. (2018) [28]	Carriers of the minor allele homozygous genotype of the two AKNA SNPs (-1372C > A and Pro624Leu) have higher possibilities of knee osteoarthritis (KOA) than carriers of the heterozygous or ancestral homozygous genotypes of each SNP	This study represents the first evidence of a potential new KOA susceptibility gene. The deregulation in the co-stimulation of the immune system cells may be the mechanism underlying such association
Piulats et al. (2018) [13]	*akna* was negatively regulated in cisplatin-resistant tumors with gains in the 9q32-q33.1 region, but not in cisplatin-sensitive tumors altered, and could be related to tumor resistance to cisplatin	AKNA could be a prognostic marker to identify cisplatin refractory tumors
Camargo et al. (2019) [18]	AKNA is a centriolar protein that localizes to the subdistal appendages of the mother centriole in neurons	Low levels of AKNA cause stem cells to remain in the stem cell niche; higher levels promote their detachment from the niche, favoring migration
Hug et al. (2019) [14]	Detected a spontaneous variant in *akna* resulting of a 4-bp deletion and a truncated variant of AKNA as the cause of a severe autosomal recessive lung disease in dogs, resembling primary ciliary dyskinesia	As an important anti-inflammatory regulator of the immune response, AKNA should be considered as a candidate gene for human patients with unexplained recurrent inflammatory pulmonary disease
Pereira et al. (2019) [15]	AKNA was related to racing performance in the quarter horse breed, with a positive regulation of transcription from polymerase II RNA promoter	AKNA could be a gene related to speed for the racing line of quarter horses
Song et al. (2019) [23]	AKNA was one of the 16 hub genes identified in head and neck squamous cell carcinoma (HNSCC) tumorigenesis	AKNA plays an important role in HNSCC tumorigenesis
Shamseldin et al. (2020) [24]	A homozygous nonsense variation in *akna* 1990C > T:p.(Glna664*) was associated to primary ciliary dyskinesia (PCD)-like disease	AKNA is proposed as a novel candidate in a lung phenotype that overlaps clinically with PCD and potentially in ciliated cell function
Wang et al. (2020) [25]	AKNA is downregulated in gastric cancer (GC). AKNA is proposed as a potential tumor suppressor, affecting EMT-related pathways including chemokines and cytokines signaling pathways. AKNA might be regulated by circTRNC18/miR-762 axis	AKNA could serve as a potential biomarker and an effective target for GC diagnosis and therapy
Zhao et al. (2020) [30]	An intronic SNP of *akna* (SNP rs10817595) is significantly associated with the risk of KOA in a sample based on the Chinese Han population	A potential link between the risk of KOA and AKNA in subjects with Chinese Han ancestry. This association signal might be explained by the upregulation of the immune response and inflammation resulting of decreased AKNA expression.

## Data Availability

Not applicable.

## Supplementary Materials

The following are available online at https://www.mdpi.com/article/10.3390/biom11111709/s1, Table S1. PRISMA Extension for Scoping Reviews (PRISMA-ScR).
